# Supplementary material for: Are carbon emissions trading and green financial instruments synergistic? -Comprehensive quantitative research based on content analysis
Source: PLoS One. 2024 Mar 7;19(3):e0298601. doi: 10.1371/journal.pone.0298601 (PMC10919601; doi:10.1371/journal.pone.0298601)
Supplement: S1 Data — (ZIP) [file pone.0298601.s001.zip › Data/地级市Z_F工作报告.zip!/─■╧─/╥°┤¿╩╨/└┤╫╘╣1⁄2╓┌║┼í╛┬φ┐╦╩2╛▌═°í┐_2016.docx]

各位代表：

现在，我代表市人民政府向大会作政府工作报告，请各位代表连同《银川市国民经济和社会发展第十三个五年规划纲要（草案）》一并审议，并请市政协委员和其他列席人员提出意见。

一、奋力攻坚，真抓实干，开放内涵式发展迈出新步伐

刚刚过去的一年，面对复杂多变的宏观经济形势和繁重的改革发展任务，在自治区党委、政府和市委的坚强领导下，在市人大、市政协的监督和支持下，全市广大干部群众迎难而上，砥砺前行，坚定不移实施“2258”工作思路，加快开放内涵式发展步伐，全市经济保持平稳较快发展。地区生产总值达到1480.73亿元，增长8.3%；地方公共财政预算收入达到171.28亿元，增长12.11%；固定资产投资1540.88亿元，增长10.6%；社会消费品零售总额达到477.63亿元，增长7.2%；城镇和农民人均可支配收入分别达到28260元和11148元，增长8.2%和8.5%；新增城镇就业6万人，城镇失业登记率控制在3.9%以内。基本完成了“十二五”期间主要目标任务，为“十三五”良好开局奠定了坚实基础。

（一）精准发力，综合施策，经济运行稳中有进、进中向好。针对经济下行压力持续加大的态势，我们在全面落实自治区“工业18条”“财税20条”等政策的基础上，结合市情，坚持需求和问题导向，深入开展“财源建设年”“改革落实年”“项目建设年”等活动，及时出台《银川市工业产业结构调整和转型升级实施方案》《关于推进工业振兴工程扶持政策》及金融“十二条”新规等，综合运用产业基金、担保和债券融资等金融工具，吸引、撬动社会资本200亿元支持优势特色产业发展。培育22家企业在新三板上市。设立先进装备制造、纺织等10支产业基金，规模达到50亿元；放大“西部担保模式”，为企业担保融资86亿元；成功发行20亿元中期票据和全国第二单20亿元滨河黄河大桥项目收益债；争取国家专项建设基金29亿元。采用PPP投资模式，建设“智慧城市”和兵沟黄河大桥项目。安排5123万元表彰奖励对银川发展做出突出贡献的企业。采取援企稳岗、降费减税、电气价优惠等“一揽子”降成本措施，取消和免征行政事业收费82项，减免税费32亿元，帮助企业降低用电成本5.3亿元。扎实开展“准备之冬、建设之春、发展之夏、收获之秋”系列活动，全市1075个基本建设项目全部开工，完成投资1410亿元，其中90个重点项目完成投资320亿元。现代纺织产业示范园“一园三区”、隆基硅年产1.2GW单晶棒和滨河、兵沟、永宁三座黄河大桥等项目加快推进，轨道轴承、晶明科技高纯氧化铝和中兴智慧城市大数据一期等项目建成投用。银西高铁获批动工。

（二）调转结合，增强动能，引领经济发展新常态。实施工业振兴工程。宁东基地、经济技术开发区等8大重点产业园区，总产值超过千亿。培育壮大能源化工、装备制造、新材料等百亿、千亿级产业集群，规上工业总产值达到1868.92亿元。建成全国最大的单晶硅棒和蓝宝石生产基地，小巨人数控机床出口欧洲，舍弗勒轴承为宝马和奥迪汽车配套，共享铸钢铸件单体成为世界最大，巨能机器人被工信部誉为“制造智能工厂的工厂”。累计发展高新技术企业51家，占全区80%以上。科技专利申请量和授权量分别达到3306件和1255件。轻工业产值同比提高2.3个点。完成西轴数字智能化热处理工艺等60个重点技改项目，淘汰水泥等落后产能56万吨。规上工业综合能源消费量同比下降3.5%；万元GDP能耗同比下降3.1％。大力发展新产业、新业态。实施“反梯度”发展战略，建成银川通航产业园暨通用机场一期以及电商谷、电商物流园等一批新产业载体和平台。神马直升机制造首批组装完成14架旋翼机。iBi育成中心跻身中国产业园创新力百强，“iBi模式”在全国推广。成功举办第二届WCA世界电竞大赛，产业规模和品牌影响力持续扩大。城市电商发展迅猛，农村电商覆盖率达60％，全市网络零售交易额达到90亿元，增长45%。“一图一网一云”智慧城市成为全国样板，荣获TMF2015年度世界智慧城市总裁奖，入选“国家信息消费示范城市”。推进服务业提档升级。实施润恒城、新华联广场等商贸流通项目110个，完成投资160亿元。银川国际美容整形中心投入运营，卡瓦心脏中心累计完成心脏疑难手术315例。新引进金融机构7家，金融业增加值占到服务业增加值的25%。文化体育旅游融合发展，军博园二期、三沙源等项目加快建设，银川韩美林艺术馆、当代美术馆开馆，水洞沟、军博园分别跻身国家5A级、4A级旅游景区，获评“中国最具特色旅游城市”。西夏陵列入国家申遗计划。成功举办银川航展等系列活动。组团参加全国首届青年运动会，取得“两金一银一铜”佳绩。加快发展“三精农业”。粮食产量实现“十二连丰”，新增设施园艺5000亩，优质水稻基地2万亩，建成标准化养殖基地30个，“菜篮子工程”基本满足市民需求，葡萄及葡萄酒庄产业发展迅猛。三大主要农作物机械化率达到91%以上，完成“三品一标”认证63个，10家企业荣获第十六届绿博会绿色食品金奖。

（三）统筹推进，夯实基础，城乡面貌深刻变化。增强规划先导作用。完成全域银川战略策划、产城一体、生态规划三大顶层设计，中心城区控规做到全覆盖，实现“三规合一”及“多规融合”，带动城乡一体发展。加快完善基础设施配套。高标准完成110国道综合整治工程，新建改造贺兰山路等21条主干道路、55条小街巷，新增城乡公路231公里，建成覆盖全域、连通周边的“1小时交通圈”。新购置626辆高配置公交车，城市公交分担率达到38.5%。全面建成覆盖市域行政村的客运网络，城乡居民出行条件极大改善。投资4.2亿元完善城乡电网基础设施。改善提升人居环境。实施棚户区改造项目21个，新建安置区150万平方米，近2万人乔迁新居。完成7个美丽城镇、15个美丽村庄建设，惠及10余万群众。持续开展主干道大整治、大绿化，完成人工造林12.4万亩，绿地率达到42%，高于全国平均水平。建成绿博园和10个市民休闲小微公园，成功申办第九届花博会。实施“蓝天工程”。整治拆除224台燃煤小锅炉，淘汰黄标车1.7万辆，空气质量四项主要指标实现“一平三降”。改造提升湖泊水系57.2万平方米，治理“龙须沟”30公里，改善沿线12.8万群众居住生活条件。深化完善全域“大城管”格局。城市市容环境卫生管理体制改革试点起步，“以克论净”深度清扫保洁和城市精细化管理水平不断提升。城管巡回法庭模式荣获“全国生态环境法治保障制度”创新事例优秀奖。餐厨垃圾改造提升项目投入运行，建成24个垃圾分类示范点，垃圾焚烧发电等资源化利用产生效益4600余万元，列入全国首批生活垃圾分类示范城市。国家卫生城市复审进入前十。入选“中国最美丽的城市”。

（四）搭建平台，连接通道，对外开放迈出扎实步伐。“三位一体”开放核心区生机勃勃。滨河新区“四城”进入了由点到面、由局部到整体开发新阶段。累计实施大环境绿化、全域5A级旅游景区等项目110个，完成投资170亿元。恒天如意科技产业城、中联重科环卫机械制造等项目加快建设，国际医疗城主体封顶，国际科教城投入运营，黄河外滩公园、薰衣草庄园等景区相继开园。综合保税区成功加入世界自由区组织，成为具有选举权的第二家中国海关特殊监管区域。懿丰葡萄酒产业园、黄金珠宝文化产业园、如意日产800吨气流纺纱等项目建成运营。获批进境肉类（水果、种苗)口岸加快建设。新增注册企业90家。临港经济区基础设施进一步完善，中玺枣业、天籁生物项目建设加快。阅海湾中央商务区30栋总部楼宇主体封顶，宁夏国际会堂、银川国际交流中心两大城市地标建成投用，西北首家国际商品保税展销中心开业。金融中心引入华融西部资产管理等126家金融机构、电子商务企业，独栋楼宇创造产值50亿元。打造“三条丝绸之路”，推进开放合作。新开通国际客运航线5条，首个城市候机楼启用，空港口岸年出入境人数突破10万大关。与郑州等丝绸之路经济带沿线城市9个海关建立区域通关一体化。丝路通B2C电子商务交易平台上线运营。成功举办TMF全球智慧城市主题峰会、银川开放高峰论坛等系列节会，签订合作项目40个，投资333亿元。全年实现招商引资实际到位资金500亿元；完成外贸进出口总额32.66亿美元。

（五）锐意改革，优化环境，发展活力进一步释放。加大简政放权力度。以打造“两优”环境为目标，政府行政职权由原来的8570项压减掉65%。审批环节再减149个，审批效率提升30%；建成“网上办事大厅”，网上审批占比达到35%。创新“三证合一”“一照一码”和“先照后证”商事管理和服务机制，“智慧政务银川模式”走在全国前列。近两年全市新登记注册企业17941户，是“十二五”前三年的1.5倍。深化国企改革。组建通联资本、新兴产业和金融控股三大国有资本投资运营平台，初步形成“四级国有资本监管模式”，实现管资产向管资本的转变。建立国有资本经营收益上缴预算管理制度。扎实推进农村改革。基本完成农村集体土地确权登记，建成永宁、贺兰农村产权交易中心，金凤区成为“全国集体资产股份权能改革试点县”。全市35个村开展农村社区化试点。建立了蔬菜、奶业风险补偿基金和农机互助保险。开展“双增双减”活动，空壳村脱壳率达到78%。其他重点领域改革稳步推进。组建全国首家人才工作服务局，建成西北人才金港等8个引才育才载体。启动实施机关事业单位公车和养老保险制度改革。稳步推进国家城市公立医院改革试点，药品零差率、先看病后付费和县乡一体化医疗卫生服务取得新进展。

（六）织网兜底，改善民生，社会事业全面发展。推进创业带动就业。健全完善全民创业、大学生就业、职业技能培训等政策体系，高校毕业生当年就业率保持在90%以上，开展技能培训10.17万人，转移农村劳动力就业11.27万人。加快完善社会保障制度，城乡居民基本养老保险、基本医疗保险实现全覆盖，基础养老金标准居西部省会城市首位。率先建立被征地农民养老保险。实行大病医保上不封顶和重特大疾病医疗救助制度。“四位一体”精准扶贫模式成效显著。圆满完成自治区下达“十二五”生态移民搬迁安置任务。打造的“闽宁扶贫”开发样板镇成为全国典型。移民地区农民人均可支配收入达到8700元，增长11.8%。推进优质教育均等化。启动政府购买普惠性学前教育服务，新建19所标准化中小学，率先在全区实现义务教育均衡发展全覆盖，建立教育质量第三方评价机制。加快医疗卫生硬件建设。新建扩建宁医大胸科医院、市口腔医院新院及社区、乡村医疗卫生服务机构17家。着力构建公共文化服务体系。新建投用市文化馆新馆及乡镇街道文化站、农民文化大院等68个，图书馆、文化馆免费向市民开放。群众性文化活动蓬勃开展。推进社会治理创新。健全完善社会风险防控预警机制，多警联勤联动快速高效处置刑事和治安案件，依法打击各类违法犯罪活动，加大和谐社区构建力度，群众安全感指数稳定在95%以上，入选全国最安全城市。被评为全国“六五”普法中期先进市。率先在全区实现信访信息系统三级联网，初信初访办结率持续保持100%。建立“党政同责、一岗双责”安全生产责任体系，整合市场监管职能，特种设备、食品药品等安全生产形势保持稳定。

（七）完善制度，优化服务，政府自身建设不断加强。制定《政府议事规则》及政府规章和规范性文件14部，依法公开8.8万余件政务信息。荣获“中国第八届政府创新优秀实践奖”。自觉接受人大及其常委会法律监督、工作监督和政协民主监督，提请人大常委会审议地方性法规草案2部，办理人大代表建议意见和政协委员提案593件。国家土地、审计和自治区巡视整改率达到100%。严格执行中央八项规定、国务院“约法三章”和区市若干规定，持之以恒纠正“四风”和“不严不实”问题，实施“电视问政、电视施政”9期，严肃查处违反“八项规定”问题18个，问责处理26人。

一年来，我们统筹推进国防动员、双拥优抚等工作，支持工会、共青团、妇联和残联等群团组织开展工作。统计、外事、侨务、仲裁、公积金、地震、气象、人防、档案、文史等工作取得新成绩。民族团结、宗教和顺、军民一家亲的局面更加巩固。

总结2015年的各项工作，困难比预料的多，取得的成效比预期要好。回顾整个“十二五”的发展变化，我们经受住了考验，付出了艰辛，创造了辉煌。这五年，银川的变化最大。我们向建设西北地区最适宜居住、最适宜创业的现代化区域中心城市迈出重大步伐，成功创建“全国文明城市”；建设的阅海湾中央商务区、滨河新区、综合保税区等功能区成为推动开放发展的新载体、新引擎，后发优势明显；“一带一路”节点城市、内陆开放型经济核心区、“碧水蓝天、明媚银川”等城市新名片成为世界记忆银川的符号。这五年，经济综合实力大幅跃升。我们直面发展困难和挑战，用中央、自治区一系列新思想、新论断指导新的实践，一切从实际出发，倡导“深、实、诚、细、严”的作风，敢于担当、勇挑重担、狠抓落实。经济总量、公共财政预算收入、固定资产投资、社会消费品零售总额分别是2010年的1.9倍、2.7倍、2.4倍和2.1倍。这五年，产业结构调整取得重大突破。我们针对经济发展倚重倚能、结构性矛盾日益突出的现实，坚定不移调结构，创新驱动转方式，聚精会神提质效，新型能源化工、高端装备制造等产业链条延伸、效益提高，现代纺织、大数据、通航产业、电竞产业等新产业、新业态发展势头强劲，旅游、金融、电商、物流等现代服务业快速发展。轻工业占比提高4.5个百分点，三次产业调整为3.9∶53.2∶42.9。这五年，群众得到实惠最多。我们在政策性减税、行政事业性收费大幅压减的情况下，民生投入逐年递增，累计投入达到794.7亿元，年均占公共财政支出的80%以上。城镇和农民人均可支配收入年均分别增长10.8%和11.9%，快于GDP增速。先后3次提高最低工资和城乡居民养老金标准，5次提高城镇职工基本养老金标准。旧城（棚户区）改造共拆迁1661.86万平方米，惠及42658户20余万人。10余万人摘掉“穷帽子”。

各位代表，五年来，勤劳智慧的银川人民，用汗水和力量、激情与拼搏，创造了银川速度和银川效率，书写了银川发展史上的精彩篇章！这是自治区党委、政府和市委坚强领导的结果，是市人大、政协监督支持的结果，是全市人民艰苦奋斗、共同努力的结果。在此，我代表市人民政府，向辛勤奋战在各条战线上的广大干部群众，向给予政府工作大力支持的人大代表、政协委员、各民主党派、工商联、无党派人士和人民团体，向驻银部队和武警官兵、公安民警，向为银川改革发展做出贡献的社会各界朋友，表示衷心的感谢和崇高的敬意!

在肯定成绩的同时，也要清醒的认识到，我市经济社会发展和政府工作还存在一些不足和薄弱环节。一是经济总量不大，在西部省会（首府）城市中排名靠后，产业优化升级、结构调整任重道远。二是“创新型银川”亟待突破，科技研发、科技支撑、人才队伍建设等方面相对滞后，缺乏重大科技项目及平台支撑。三是对外开放水平还不高，开放平台开放通道和辐射源建设还处在培育阶段，与内陆开放型经济试验区核心区不相匹配。四是城乡发展不平衡，万元生产总值能耗等8项全面小康指标实现难度较大。五是政府职能转变还不到位，公务员队伍中不敢担当、不善作为、消极畏难的现象还程度不同地存在。对此，我们一定要高度重视，采取有效措施，切实加以解决。

二、科学谋划，创新理念，率先建成全面小康社会

“十三五”时期，是全面建成小康社会的决胜期，是全面深化改革的攻坚期，更是我市创新发展、转型追赶的关键期。

政府工作总体要求是：高举中国特色社会主义伟大旗帜，全面贯彻党的十八大和十八届三中、四中、五中全会精神，深入贯彻习近平总书记系列重要讲话精神，坚持“四个全面”战略布局，坚持“五大发展理念”，认真贯彻自治区党委十一届六次、七次全会和市委十三届六次全会精神，以融入“一带一路”战略为主线，以提高发展质量和效益为中心，加快形成引领经济发展新常态的体制机制和发展方式，统筹推进经济建设、政治建设、文化建设、社会建设和生态文明建设，加快开放内涵式发展，在全区率先全面建成小康社会，加快现代化、国际化进程，建设丝绸之路经济带重要节点城市，打造最适宜居住、最适宜创业的区域中心城市。

主要目标：在提高发展质量的基础上，经济增速高于全国和全区平均水平，年均增长8%以上，到2020年地区生产总值突破2000亿元；城乡居民人均可支配收入年均分别增长9%和10%；地方公共财政预算收入年均增长8.5%；全社会固定资产投资年均增长10%以上；社会消费品零售总额年均增长8%以上；主要约束性指标完成自治区下达任务。在综合经济实力增强的同时，转型升级实现新突破、新型城镇化达到新水平、改革开放开创新局面、生态文明建设取得新成效、人民生活得到新改善、社会文明程度得到新提高。

各位代表，我们作为欠发达地区加快发展是第一位的，实现全面小康是人民的期盼和愿景，也是我们每个人应该肩负的光荣责任和庄严使命。从国际国内看，当前，国际金融危机深层影响还将持续，世界经济在深度调整中仍然延续疲软复苏的态势，但新一轮科技革命和产业变革蓄势待发，孕育着新产业、新业态、新模式，将迎来新的发展空间和突破。我国经济发展长期向好的基本面没有变，正在由原来注重发展速度、规模扩张向提高发展质量和效益并重转变。从全区和我市看，随着国家深入实施西部大开发、“一带一路”倡议，以及我们多年来的发展基础，干部群众齐心协力，干事创业氛围更加浓厚。

站在新的历史起点上，放眼未来，落实好“十三五”规划蓝图，率先决胜全面小康，我们必须坚定不移地全面贯彻落实“五大发展理念”，努力做到一体坚持、一体贯彻，以理念领先，带动发展争先，实现有质量，有效益，没水分，可持续的发展。

一是坚定不移落实创新发展理念，开创转型升级新局面。把创新作为引领发展的第一动力，以创新扩大有效需求、释放消费潜力、带动产业转型升级。加快“创新型银川”建设。设立10亿元科技创新发展基金，力争撬动企业、社会资本投入技术研发经费达到40亿元。围绕我市传统产业改造和新兴产业发展，实施一批重大科技专项，突破一批优势特色产业关键技术，掌握话语权。把大众创业、万众创新融入各领域各环节，借助“互联网+”，搭建众创、众包、众扶、众筹四众平台和协同机制，让四众拾柴火焰更高。大力推进金融创新，全力打造西部地区金融中心。力争到2020年，科技进步贡献率达到55%；轻重工业比达到30：70；规上战略新兴产业占到工业增加值15%。

二是坚定不移落实协调发展理念，形成统筹推进新格局。以宁夏空间发展战略规划为抓手，优化生产力布局，加快新型工业化、信息化、城镇化、农业现代化进程。增强区域发展的协调性，加强与周边基础设施互联互通，推动全域发展，支持发展壮大县域经济，到2020年县域比重占全市GDP的50%以上。增强城乡发展的协调性，完善城市治理体系，提高城市治理能力；加快公共资源向农村配置，常住人口城镇化率达到78%以上。增强物质文明和精神文明建设协调性，巩固发展文明城市创建成果，到2020年各县（市）区全部进入全国县级文明城市创建行列。

三是坚定不移落实绿色发展理念，彰显宜居宜业新优势。坚守土地、资源、环境三条底线，强化生态建设和环境保护，发展低碳循环经济，倡导绿色消费生活方式，推进生态文明建设。完善生态补偿机制，实施大气、水、土壤污染防治行动计划。整治搬迁中心城区内所有重污染企业，力争单位能耗降到同行业最低水平。坚持西防水、中理水、东治水，留住天上水，用好黄河水，保护地下水，多元利用中水。坚持中治黄河、东治沙，灌区林网高标准化，渠系公路百里林荫化，城市湖泊水系园林化。到2020年森林覆盖率达到18%，人均拥有公园绿地面积达到17.5平方米。

四是坚定不移落实开放发展理念，构筑内陆开放新高地。主动融入国家“一带一路”战略，坚持全面开放与重点突破兼顾，引进来与走出去结合，引资与引技引智并重，对内开放与对外开放协调。积极推动宁东基地、滨河新区、综合保税区、现代纺织产业园、河东机场、临港经济区“六位一体”融合发展，申建国家级新区。依托综合保税”。完善提升“三条丝绸之路”建设水平，加快推进银川大开发向银川大开放转变。

五是坚定不移落实共享发展理念，实现人民福祉新提升。坚持把实现好、维护好、发展好最广大人民根本利益作为发展的根本目的，切实保障和改善民生，实现基本公共服务全覆盖。坚决打赢脱贫攻坚战，率先在西北省会城市脱贫。分配好发展的蛋糕，健全科学的工资正常增长机制，确保2020年城乡居民人均收入较2010年翻一番。不断增强社会保障和兜底能力，改善提升教育卫生、文化体育、养老服务等社会事业发展水平，切实增强人民群众的幸福感和满意度。

三、创新发展，转型追赶，奋力实现“十三五”良好开局

今年是实施“十三五”规划的起始之年，是全面建成小康社会进入决胜阶段的重要一年，也是推进结构性改革的攻坚之年。做好今年的工作至关重要。

今年的主要预期目标是：地区生产总值增长8%；全社会固定资产投资增长10%；公共财政预算收入增长8.5%；社会消费品零售总额增长8%；城乡居民人均可支配收入分别增长9%和10%；居民消费价格指数控制在3%左右；城镇新增就业人数4.5万人，城镇登记失业率控制在4.5%以内；主要约束性指标完成自治区下达任务。

（一）坚持项目带动，量质并举，持续扩大有效投资。既注重投速、投量，更注重投向、投效，以项目保投资、稳增长、促转型、惠民生。

加快重点项目建设。协助抓好自治区河东机场三期扩建、银西高铁、银川至宁东轻轨、京藏高速改扩建、神华煤制油、宁夏美术馆等项目。着力抓好全市100个重点项目，完成投资400亿元。实施180个基础设施项目。规划建设银川旅游环线轻轨、火车东站综合客运枢纽及银川至巴彦浩特、乌海、鄂尔多斯城际铁路。统筹推进滨河新区、综合保税区、阅海湾中央商务区、科技园等重大基础设施建设项目。实施300个产业项目。开工建设共享模具智能工厂、宝丰1GW光伏发电等项目，加快推进隆基硅3GW切片、西轴高端轴承产业化基地等项目，建成如意高档服装、中联重科环卫机械制造、银峰铝挂车等项目。实施75个民生项目。抓好银川市儿童医院、特殊教育中心迁建等项目。拓宽项目融资渠道。采用PPP模式和政府购买服务等方式，实施市政、交通、生态等领域47个项目，完成投资510亿元。归并财政资金25亿元，做大西部担保、产业基金规模，支持企业对接资本市场，扩大股权、债券和票据等直接融资模式。实行财政性资金存储竞争性管理，引导地方金融机构开展离岸金融、基金配资、资管计划等援企共赢业务，支持实体经济发展。完善项目推进落实机制，健全“督查、考核、服务”体系，继续推行领导包抓重点项目责任制，建立台账，倒排工期，挂图作战。深入研究国家经济政策、产业布局、投资方向，谋划储备一批重大基础设施、产业发展、公共服务、生态环保等项目。坚持招商造商引资引智并举。上争项目资金150亿元，同比增长15%。突出精准招商和定向招商，加快完善企业、项目、技术、人才等落地优惠政策，健全基金引导、高比例配资等造商引资机制，开展招大引强对接服务行动，力促北京四方太阳能电池及光伏电站、丝路国际合作产业园等项目落地。力争引进500强企业5家，完成招商引资项目到位资金550亿元。

（二）坚持创新驱动，增调同步，推进产业转型升级。围绕产业链布局创新链，依托创新链提升价值链，推动产业由中低端向中高端迈进，打造银川经济“升级版”。

实施创新驱动战略。加快推进宁夏沿黄经济带科技创新改革试验区核心区建设。突出政府创新引导。推进iBi育成中心与银川科技园融合发展。抓好中科院银川中心、清华大学绿色节能建筑、宁煤研发中心等11个研发机构和科技成果转移转化中心建设。实施“两个国家级开发区”提档升级工程，支持有条件的县（市）区谋划建设新的科技创新平台。推动金融与科技深入融合，争创国家第二批科技与金融结合试点城市。突出企业创新主体。落实国家研发费用加计扣除等政策，健全完善高新技术企业扶持、科技专利资助、创新后补助等激励机制，实施高新技术企业、科技型中小企业数量倍增行动计划，力争全年新增48家。深入实施中小企业知识产权战略推进工程，专利申请量、授权量分别增长15%和20%，加强知识产权保护，营造良好的“双创”生态。突出人才创新支撑。全面推进人才管理改革试验区建设，建立与贡献相匹配的创新收益和风险补偿机制。实施“三项人才工程”，加快筹建银川人才产业园和欧、美等国际人才联络工作站，启动实施国际人才公寓项目，搭建一批高水平院士工作站、重点实验室、工程技术研究中心和“众创空间、众创工厂”，引进培养阿语、金融等各类急需紧缺人才1000名。

实施优势主导产业带动工程。以“中国制造2025”和“互联网+”行动计划为引领，促进主导产业优化升级。大力发展现代纺织产业。加快实施宝塔石化120万吨/年PTA及配套原料、现代纺织产业示范园“一园四区”项目，推动煤化工与现代纺织融合发展，打造纺织全产业链和产业集群，力争纺织产业产值增长25%，轻工业比重提高2个百分点。做强装备制造和新材料产业。支持小巨人数控机床、共享铸钢、西北轴承等高端装备制造业，加大科技攻关及核心技术突破，推动向智能化、无人化转型。支持隆基硅、工业蓝宝石、碳化硅等企业，由初级原料加工向光伏材料切片等中高端产业链延伸，从行业“单打冠军”向“集团军”迈进。做优葡萄及葡萄酒庄产业。继续完善贺兰山东麓酿酒葡萄产业基础设施配套，加快推进葡萄标准化种植和列级酒庄建设，打造集葡萄种植、酿造、营销、品牌、文化旅游等全产业链。做活文化旅游产业。攻坚西夏陵申遗，抓好西夏博物馆迁建，推进非遗产业化。完善提升“两带一轴”景区景点服务功能，打造国家十大旅游休闲示范城市。开通丝路旅行网，办好中美旅游年系列活动，加强与美国、韩国等国际旅游合作。放大卡瓦心脏手术、医学整形美容、干细胞临床应用等医疗健康资源，吸引更多的游客来银旅游休闲、健康养生。

实施新产业新业态培育工程。坚持“反梯度”发展，“零度承接”产业高端，一步到位发展数据信息、游戏动漫、通用航空、电子商务等新产业、新业态。加快信息消费和宽带中国示范城市建设。推动大数据在智慧政务、智慧交通、智慧环保等10大领域的研究与应用，率先在全国实行大数据信息开放。举办第二届TMF智慧城市全球峰会，提升iBi育成中心轻资产、新业态集聚研发能力，催生智慧产业链创新与信息产业聚集发展，支持“智慧城市”上市融资。打造世界电竞之都。制定出台电竞产业发展规划和扶持意见，规划建设电竞大厦，继续办好WCA世界电竞大赛，推进全年大中小赛事常态化。支持圣地等国际国内游戏开发、动漫软件、赛事策划、游戏体验等企业和机构，创新游戏商业模式，打造游戏全产业链。抓好银川通航产业园招商运营。深化与北汽通航、斯洛文尼亚蝙蝠飞机制造等企业战略合作，开展飞机组装、拆解、航空器维修等业务，引进培养一批通航飞行、维修、空管等人才，开发通航文化体验、空中揽胜、航空运动竞赛等产品，打造通航全产业链集群。推进电子商务示范城市建设。制定电商产业发展意见和专项规划，支持本地电商企业与国内外知名电商和第三方支付平台战略合作，推动传统企业转型线上、平台企业布局线下融合发展，鼓励社区Ｏ2Ｏ电商发展新兴配送模式，农村电商开发“私人定制”生产模式，力争全年网络交易额增长45%以上。

实施传统产业改造提升工程。推动工业化和信息化深度融合。支持能源化工、装备制造、生物发酵等传统产业，对标国际国内同行标杆，推进技术革新和改造提升，增强核心竞争力。全年实施技改项目50个，支持宝丰、宝塔石化等骨干企业进行战略合作或兼并重组，打造行业旗舰。支持灵武市建设“城市矿产基地”，大力发展循环经济。力争规上工业增加值增长8.5%。抓好国家服务业综合改革试点城市建设。加快实施108个重点商贸物流项目，抓好大阅城、铂尔曼酒店等“双十工程”建设，举办房车博览会、智能制造设备（技术）等品牌会展活动。加快推动润恒、公铁物流园等项目建设或运营，支持骨干物流企业建设标准化仓储、物流信息化服务平台，探索城市共同配送新模式。积极推动家庭和养老服务业发展。规范社会中介机构运行，完善市场经济体系。力争第三产业增加值增长8%。大力发展都市型现代农业。以“三精农业”为抓手，加快建设国家级现代农业示范区。做精做强设施园艺、草畜等五大优势特色产业，重点培育长枣、有机水稻、“塞上花卉”等叫响全国农字号品牌。推进互联网+农业，支持农字号龙头企业、新型农村经营主体建设综合信息服务平台和精品农业示范基地（园区），带动农业市场化、倒逼标准化、促进规模化、提升品牌化。探索建立水产品风险补偿基金和保险基金。建立完善农产品质量安全可追溯体系，力争特色农产品加工转化率达到75%以上，农业增加值增长5%以上。

（三）坚持开放带动，先行先试，拓展内陆发展新空间。全面落实“开放宁夏20条”和开放银川建设《意见》，发展更高层次、更高水平的内陆开放型经济。

培植开放载体，打造内陆开放新高地。滨河新区要提升“四城”建设水平。主动承接宁东基地产业链延伸和服务配套功能，推进区域互联互通，抓好宝丰、中铁二十三局200万平方米职工安置住房及景城、横城整体商业开发项目。加快实施恒天如意科技产业城、大数据中心二期和天山海世界等80个项目，完成投资120亿元。依托国际科教城、国际医疗城，重点引进一批国际高端学府、医疗和养老机构。综合保税区要提升外向型经济发展水平。加快二期全面建成封关运行。启动实施宁夏医科大总院生物科技等四大项目，着力提升黄金珠宝、葡萄酒、现代纺织等主导产业运营能力，高标准建成宁夏进境肉类（水果、种苗）指定口岸，争取获批整车、水产品、粮食、药品等指定口岸，全年实现外贸进出口总额30亿美元，增长35%。复制国内自由贸易区创新政策，开展选择性征税和一般纳税人资格试点，增强服务外向经济发展能力。支持有条件的县（市）区设立保税监管场所。临港经济区要主动融入区域一体化发展，加快发展航空配套服务、电商物流等特色产业。阅海湾中央商务区要提升商气、集聚人气。积极争取设立外国领事代办处、官方民间合作交流办事处等机构，规划建设帕西姆免税店，力争在对阿人文交流、人才培养和金融合作等方面取得突破。加快实施金银街“迪拜城”等12个项目，力促工商银行营业大楼等22个总部楼宇竣工或开业，引进新华人寿保险、中国微电影集团等100家企业入驻，做大保税展示交易中心、CBD金融中心等主导产业，力争实现产值200亿元。

加快完善开放大通道、大通关体系。陆上丝绸之路，积极融入“丝绸之路经济带”区域通关一体化，巩固提升与青岛、天津、乌鲁木齐等口岸通关协作，推进与乌力吉等口岸合作，完善银川陆港功能。空中丝绸之路，放大银川国际航空物流区位优势，规划建设国际航空物流园，打通机场物流绿色通道。开辟更多国际国内航线和航班，实现80%省会城市直飞。争取国际中转旅客72小时过境免签、境外旅客购物离银免税等政策落地。网上丝绸之路，对接国家“信息丝路”计划，完善拓展综合保税区跨境电商平台功能，建成宁夏电子口岸平台一期，争取获批跨境电商保税进口资质，鼓励跨境电商企业在迪拜等地建设与综合保税区联动的海外仓，探索B2B业务模式。

推进全方位开放合作。深化与丝路经济带沿线、东北、东南亚国家及港澳台等地区的合作和经贸往来。更加主动承接长三角、珠三角、京津冀等发达地区产业转移和创新成果转化，务实推进陕甘宁蒙毗邻城市合作。实施优进优出计划，引导外资通过股权转让、增资扩股、合资经营与本土企业开展战略合作。经济技术开发区要发挥示范带动作用。加快建设“银川卫星数据标校综合试验场”，打造天基丝绸之路。支持有条件的企业走出去，参与国家建设阿曼、沙特等境外合作产业园，并购国外一些知名品牌和先进技术，形成参与国际分工的银川方阵。培养和引进一批熟悉国际通行规则和国际惯例的涉外人才，开展对阿合作研究和应用。

（四）坚持改革引领，重点突破，增强发展内生动力。持续推进制约发展的重要领域和关键环节改革，使改革红利转化为加快发展的强大动力。

推进三大供给侧结构性改革。化解过剩产能。制定出台指导意见和定补政策，对产品有市场、有效益但暂时遇到困难的企业积极帮助脱困发展，对仍具有成长潜力的企业继续给予支持，对停产半停产、连年亏损、严重资不抵债、靠补贴和银行续贷过日子的“僵尸企业”，排队“出清”。化解过剩产能和淘汰落后产能10万吨。支持园区开展“腾笼换鸟”，让优质的土地资源发挥更大效益。加快实施西夏区老工业基地搬迁改造。化解房地产库存。出台促进房地产健康发展的意见。全面取消商品房限购条件，释放改善性住房需求。政府暂停新建保障性住房，通过回购商品房用于安置棚户区改造居民、保障低收入群体住房。探索农民宅基地与农民住房公积金制度和购房补贴挂钩等政策措施，鼓励农民进城购房落户。加快培育住房租赁市场，鼓励发展专业化企业，探索“薄利多租”的住房商业模式。降低企业成本。落实国家税费优惠政策，延长土地使用税优惠时限；提高技能培训补贴标准，减低企业住房公积金缴付比例，精简归并“五险一金”；研究制定物流补贴政策；积极推进纺织、信息产业等大用户电力直接交易；进一步清理规范中介收费，支持各县（市）区向行政“零收费”时代过渡。

推进四项重点领域改革。继续简政放权。建立并公布政府部门“三个清单”，继续推进行政审批标准化、信息化，实施“部分审批改备案”，年内再取消、下放一些审批事项；探索“网上直办、同城通办”的审批新模式，加强事中事后监管和精细化服务。深化“多证合一、一照一码”商事制度改革。打造全国审批程序最规范、审批时限最短、政务服务最优的省会（首府）城市。深化财税体制改革。推进全口径预算管理制度，严控政府债务规模。大力推进金融商业模式、服务产品创新，着力发展金融租赁、互联网金融和第三方财富管理等新业态，促进P2P、股权众筹等新型金融业态规范发展，组建地方性寿险和金融租赁公司。探索金融主体信用评级体系和“黑名单”制度，严厉打击非法集资、信用违约等违法行为，优化金融环境。力争金融增加值增长15%。深化国企改革。开展政府经营性国有资产集中清理行动，实行国资集中监管，整合资源做实滨投、房投、体投、文投等行业投资公司资产。支持国有资本运营公司与央企、外企、民企开展战略合作或兼并重组。实施中小非公企业成长倍增计划，有针对性地解决好非公企业融资、人才、用工等突出问题，力争非公经济产值占生产总值的比重达到60％以上。推进农村改革。开展“双增双减”活动，壮大村集体经济。加快农村产权制度改革，实行农村住房、宅基地 “两证合一”，设立土地流转风险保障基金，推广泰信、锦旺融资模式，探索农村宅基地使用权、房屋等抵押贷款办法，打造全产业链金融服务。推进户籍制度改革。

（五）坚持协调发展，统筹城乡，提升“两宜”城市建设水平。认真落实《宁夏空间发展战略规划》，优化市域空间、基础设施和生产力布局，在加强薄弱领域中增强发展后劲。

推动全域协调发展。立足加快建设银川、吴忠、宁东为主的大银川都市区发展定位，加强总体城市设计，启动《城市近期建设规划（2016—2020）》《海绵城市建设规划》等编制工作，深化“多规融合”。按照产城一体思路，支持各县（市）区、各功能区围绕主业，突出重点，形成功能互补、差异化定位，协调协作发展模式。加快推进贺兰、永宁与银川主城区同城化发展步伐，促进灵武、滨河新区、综合保税区、临港经济区与中心城区深度融合，推动“西夏突破”取得新进展，拓展全域银川发展空间。

着力完善城市功能。加快实施火车站综合交通枢纽和黄河航运二期工程，新建续建宝湖路跨艾依河、沈阳路下穿阅海隧道及上海路等道路桥梁工程，力促滨河、永宁、兵沟3座黄河大桥及连接线工程全面建成通车。加快实施“地下管网一张图”，重点推进大连路、怀远路等8条主干道路地下综合管廊建设，力争进入全国第二批综合管廊试点城市。新扩建第八、第九水厂。改造升级一污、二污等5个污水厂，力促七污、九污建成投用。启动实施解放西街危房（棚户区）改造工程。完成老旧小区既有建筑节能改造300万平方米。

扎实推进“美丽乡村”建设。加快农村“五改”工程，实现水电路气房光纤“六到农家”，加快农村社会事业发展，做到村卫生室、文化活动室、商业网点，农村电商、粮食超市、农机作业公司等十到位。高标准建设镇北堡红酒小镇等3个美丽小城镇和10个美丽村庄。启动城乡环境综合整治“三年行动计划”，扎实做好城乡违法建设管控工作，实现90%以上行政村垃圾有效处理，全面提升农村人居环境。

加强城市精细化、智能化管理。加快实施数字城管二期、垃圾分拣示范中心项目。改造20座智能化小型垃圾中转站，实行垃圾直运，推动垃圾分类管理立法。推广“以克论净”环卫深度保洁市场化试点。完善智能交通系统，优化区域大中小路网和公交线设置，建成6个公共停车场，新增4000个停车泊位，缓解市区局部交通拥堵和“停车难”等问题。

（六）坚持生态优先、绿色发展，持续改善人居环境。深入开展“美丽银川行动”，打造“碧水蓝天、明媚银川”城市名片。

抓好绿化美化。加快实施艾依河生态环境综合治理、贺兰山东路延伸及城市出入口等主要节点绿化景观提升和生态建设工程，新建10个小微公园，新增绿化面积500公顷，整治湖泊湿地1.1万亩。加快推进绿博园建设，筹备第九届中国花卉博览会。开展铁腕治污。强力实施“蓝天工程”，加快完善城市供热规划，启动宁东余热回收利用项目，整治拆除20蒸吨以下燃煤锅炉，力争集中供热率提高到75%。加大涉煤、涉油行业监管力度，深度治理城市扬尘、工业废气等空气污染，全面禁止黄标车上路。规划建设“大气超级站”，实时监测和解析PM2.5等空气污染源，有效治理雾霾。深入推进龙须沟治理“三年行动计划”，彻底整治二排沟等市域河道黑臭水体。加强生态保护。严守生态红线，提高项目环境保护准入门槛。健全环境信息公布、企业环保失信追责和环境损害赔偿制度。推进水生态试点城市建设，加强水源地保护，关停一批市域工业自备井。“零容忍”依法打击生态环境违法犯罪。倡导低碳生活。加快完善城市慢行系统，新增一批自行车租赁点和慢行通道，规划建设新能源（电动）汽车充电设施50个，再增加一批绿色环保公交车辆，积极创建国家第二批公交都市，倡导群众绿色低碳出行。

（七）坚持为民惠民，共建共享，提高公共服务能力和水平。保障和改善民生，政府责无旁贷。公共预算支出80%用于民生，重点办好20件民生实事，努力让群众有更多“获得感”。

全力打赢脱贫攻坚战。加快出台在西北省会城市率先脱贫的实施意见，扎实开展点对点服务、一对一救助、多对一帮扶的“点穴式”精准扶贫，不脱贫不松劲、不脱钩。抓好闽宁产业城、闽宁扶贫产业园及特色种养殖扶贫园区建设，培育发展13家劳务中介组织，免费开展贫困户劳动力职业技能和实用技术培训2万人次以上。力争移民地区人均可支配收入增长13%以上。依托滨河新区国际科教城，免费开展贫困户“两后生”职业教育3000人。推行扶贫开发政策与农村低保制度有效衔接，将丧失劳动能力的建档立卡贫困人口全部纳入农村最低生活保障政策兜底范围，力争23个贫困村全部脱贫，建档立卡贫困人口减少1万人以上。

推进创业带动就业。继续优化创业就业政策和环境，搭建一批全民创业孵化基地，培育小企业900个，培养小老板1600个。高度关注经济下行企业职工稳定就业和再就业，重点抓好高校毕业生、农村富余劳动力和困难群体的就业。开展创业就业培训1万人次，帮扶困难群体就业3000人。完善农民工工资支付保障制度，严厉打击恶意欠薪行为，保障农民工合法权益。

构建更加完善的社保体系。推进社会保险“五险合一”经办体制，完成机关事业单位养老保险制度改革，抓好城镇职工、城乡居民和被征地农民养老保险转移和接续。整合建立城乡居民基本医疗保险制度，深入推进总量控制下按病种分值结算医疗保险费用制度。完善救助体系，实施救助标准动态调整机制，兜底保障特困群众生活。实施爱心护理院二期、10个农村幸福院等项目，开展医养结合试点。争创全国七星级慈善城市。

让群众享有公平的教育。新建续建北方民族大学附属中学、华兴世纪希望小学等74个教育项目。鼓励普惠性幼儿园优质发展，深化义务教育均衡发展。试点普通高中免费教育，加强职业学校特色和骨干专业建设，深化产教融合、校企合作办学，支持和规范民办教育发展。推进“梯级”名师培养工程，提高偏远地区乡村教师待遇。开展国际教育交流，办好丝绸之路经济带沿线省会城市教育年会。全面推进教育质量综合评价改革实验工作。

提供更好的卫生计生服务。抓好市第一人民医院全科医师培训基地等8个项目建设。稳步推进城市公立医院综合改革，推行市属医院人事薪酬制度和院长年薪制，全面推进分级诊疗和城乡医疗一体化。规范化培训100名全科医师，培养100名专业技术骨干和100名学科带头人。扶持和规范社会办医，完善医师多点执业制度。落实重大疾病防控，有效应对突发公共卫生事件。加强与俄罗斯、美国、韩国等国际医疗合作。支持发展中医事业。坚持计划生育基本国策，健全完善计划生育利益导向机制，组织实施全面二孩政策，着力提升人口素质。

推动文化和体育事业发展。加快实施银川媒资基地、中心图书馆等项目建设，建成公共文化数字服务平台。深化文化体制改革，推进政府购买基本公共文化服务，丰富群众文化生活。组建文化产业联盟，发展骨干文化企业和创意产业。开展与韩国、马来西亚等国际文化交流，精雕细琢《月上贺兰》《梦回一千零一夜》等原创民族舞剧，力争推向国际市场。办好中国文化馆年会。倡导全民阅读。实施全民健身计划，大力发展体育事业。

加强和创新社会治理。加快构筑立体化社会治安防控体系，全面推进“平安银川”建设。“1·05”公交纵火案，令人痛心，教训深刻，我们要举一反三，深化矛盾纠纷排查化解，有效预防和坚决打击各类违法犯罪。开展“安全生产年”活动，加强道路交通、食药安全等重点领域专项整治，及时消除各类公共安全隐患，确保群众生命财产安全。启动“七五普法”。深入推进逐级走访和诉访分离，引导群众依法表达诉求。做好仲裁、法律援助、人民调解等工作，依法维护群众权益，化解社会矛盾。巩固深化双拥军民融合发展成果。创建全国民族团结进步示范市。

各位代表，今年的发展形势更为严峻、经济下行压力更大，面临的困难更多。信心比黄金更重要！越是在爬坡过坎的时候，越要坚定信心。各级政府及广大公职人员都要增强前景自信、优势自信、创业自信，进一步解放思想、创新实干，保持奋发向上，锐意进取的精神状态，拧住关键点，牵住“牛鼻子”，踢开“拦路虎”，打通“脑梗阻”，以干部的工作高度，提升银川的发展高度，以干部的辛苦指数，换取群众的幸福指数。

一要依法行政。牢记法律红线不可逾越、法律底线不可触碰，切实学法、守法、用法。严格按照法定权限和程序履行职责，自觉接受人大及其常委会的法律监督、工作监督和政协的民主监督，主动接受社会监督。严格执行政府议事规则、重大事项请示报告制度，认真落实重大行政决策公众参与、专家论证、风险评估、合法性审查、集体讨论决定等法定程序，提高行政科学、民主、依法决策水平。全面推进综合执法，开展“两随机、一公开”活动。

二要勤政为民。严守“六大纪律”，坚决贯彻执行市委各项决策部署。坚持走群众路线，巩固拓展“三严三实”专题教育成果，深化干部挂钩基层单位等活动，问政于民、问需于民、问计于民，对重大民生事项实行承诺制，努力使政府服务更加符合群众愿望。全面推进政务、财政预算、公共资源配置、重大项目建设等方面的信息公开，完善新闻发布制度，充分利用微博、微信等新媒体，及时传递政务信息，回应社会关切。

三要实干担当。政府全体成员要自觉践行“严字当头、干在实处”的要求，强化抓重点、抓关键工作机制，重大事项层层签订责任书，勤政敬业，务实作为，以目标倒逼责任，以时间倒逼进度，以实绩倒逼办法。进一步完善政府绩效考核办法，表彰奖励实绩突出的，约谈诫勉工作不力的，曝光追责不为乱为的，确保政令畅通、落实到位。

四要清廉从政。深入贯彻“中央八项”规定精神和区市若干规定，严格落实“两个责任”，认真履行“一岗双责”，把制度的笼子扎得更紧。保障和支持审计机关依法独立行使监督权。强化行政监察，不断完善工程建设、招标投标、土地出让、政府采购、产权交易等领域制度。加大违纪违法案件查办力度，严肃查处发生在群众身边的腐败问题，做到有腐必惩、有贪必肃，努力实现干部清正、政府清廉、政治清明。

各位代表、同志们：蓝图绘就正当乘风破浪，任重道远更需一马当先。让我们在自治区党委、政府和市委的坚强领导下，凝聚全市人民的智慧和力量，以更加饱满的激情、更加昂扬的斗志、更加务实的作风，奋力打好“十三五”开局之年的攻坚战，为建设美丽银川而努力奋斗！
